# Supplementary material for: Drosophila pain sensitization and modulation unveiled by a novel pain model and analgesic drugs
Source: PLoS One. 2023 Feb 16;18(2):e0281874. doi: 10.1371/journal.pone.0281874 (PMC9934396; doi:10.1371/journal.pone.0281874)
Supplement: S1 Fig — (A, B) Aversive rolling response of md-TRPV1(3) larvae. A capsaicin-soaked brush contacts the abdominal segment five; rolling within 10 seconds was counted as response. n = 30 for each capsaicin concentration. Error bars indicate ±SEM of more than three independent experiments. w1118 larvae were used as a control. Paired t-test, *** P < 0.001 for no capsaicin vs 20 mM capsaicin. (C) A confocal image of the mouthpart of a md-Gal>UAS-mCD8GFP fly stained with anti-GFP antibodies to indicate presence of md neurons. md-TRPV1(3) denotes one copy of md-Gal4 and 3 copies of UAS-TRPV1. (PPTX) [file pone.0281874.s003.pptx]

## Slide 1
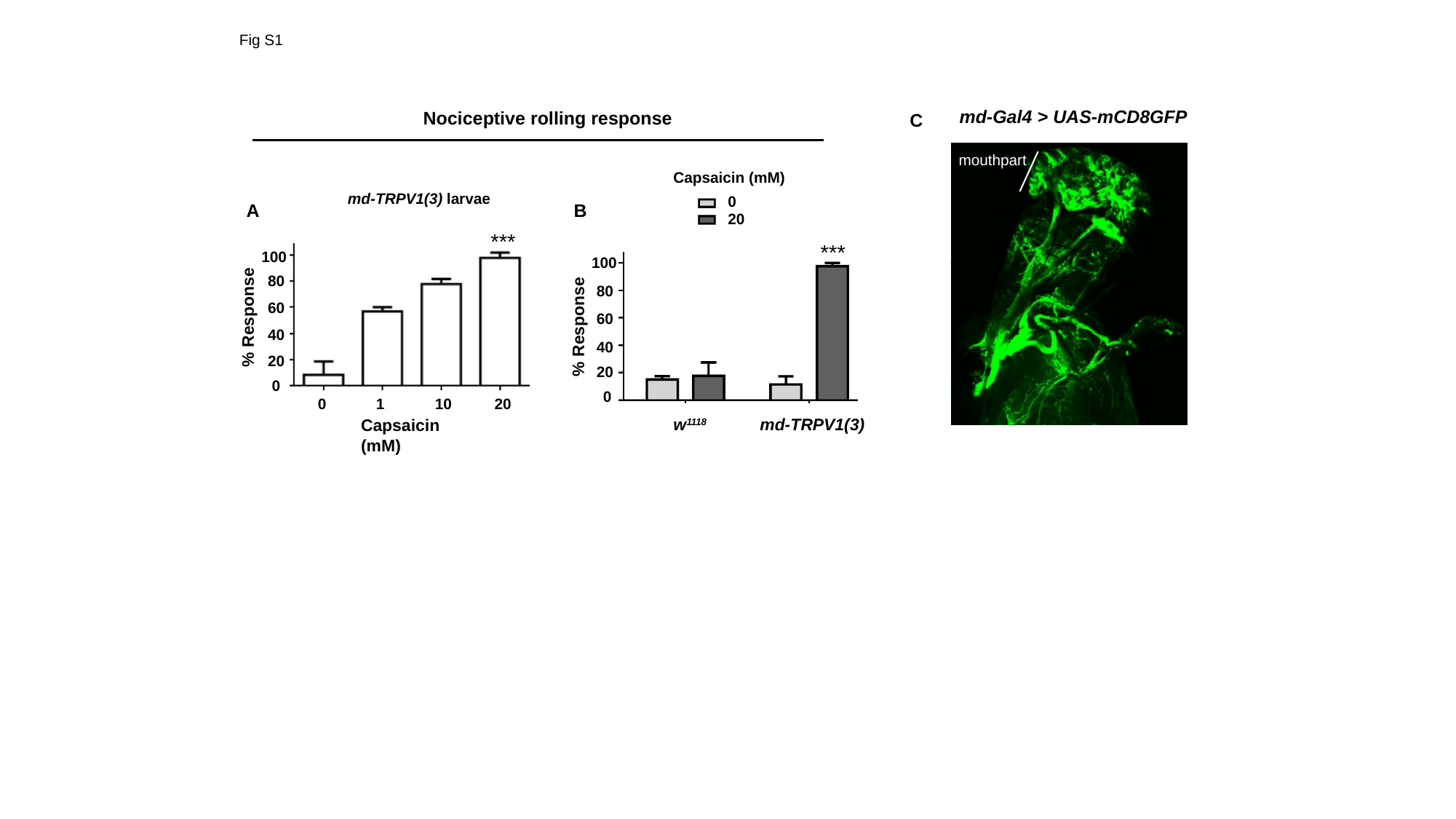

Fig S1
md-Gal4 > UAS-mCD8GFP
Nociceptive rolling response
C
mouthpart
Capsaicin (mM)
md-TRPV1(3) larvae
0
A
B
20
***
***
100
100
80
80
60
% Response
60
% Response
40
40
20
20
0
0
10
20
0
1
w1118
md-TRPV1(3)
Capsaicin (mM)
