# Supplementary figures and images for: Drosophila pain sensitization and modulation unveiled by a novel pain model and analgesic drugs
Source: PLoS One. 2023 Feb 16;18(2):e0281874. doi: 10.1371/journal.pone.0281874 (PMC9934396; doi:10.1371/journal.pone.0281874)

## Slide 1
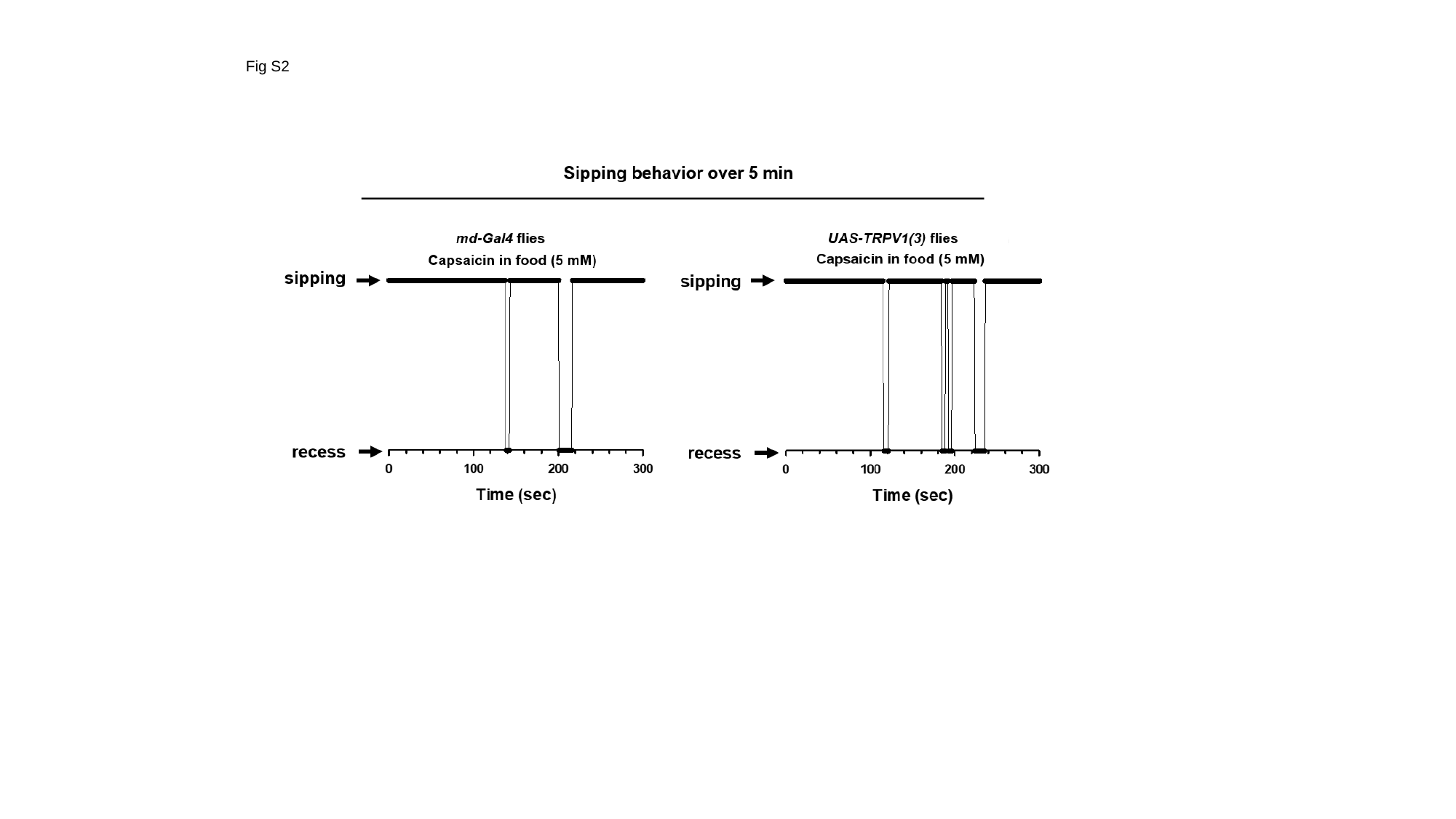

Fig S2

Supplement: S2 Fig — Hungry (18 hours starved on water-soaked filters) control (md-Gal4 and UAS-TRPV1(3)) flies were offered capsaicin (5 mM)-containing food. The flies continuously sipped for longer than five minutes. Representative behaviors are shown. Five flies exhibited similar behaviors. Five-day-old males were used. (PPTX) [file pone.0281874.s004.pptx]

## Slide 1
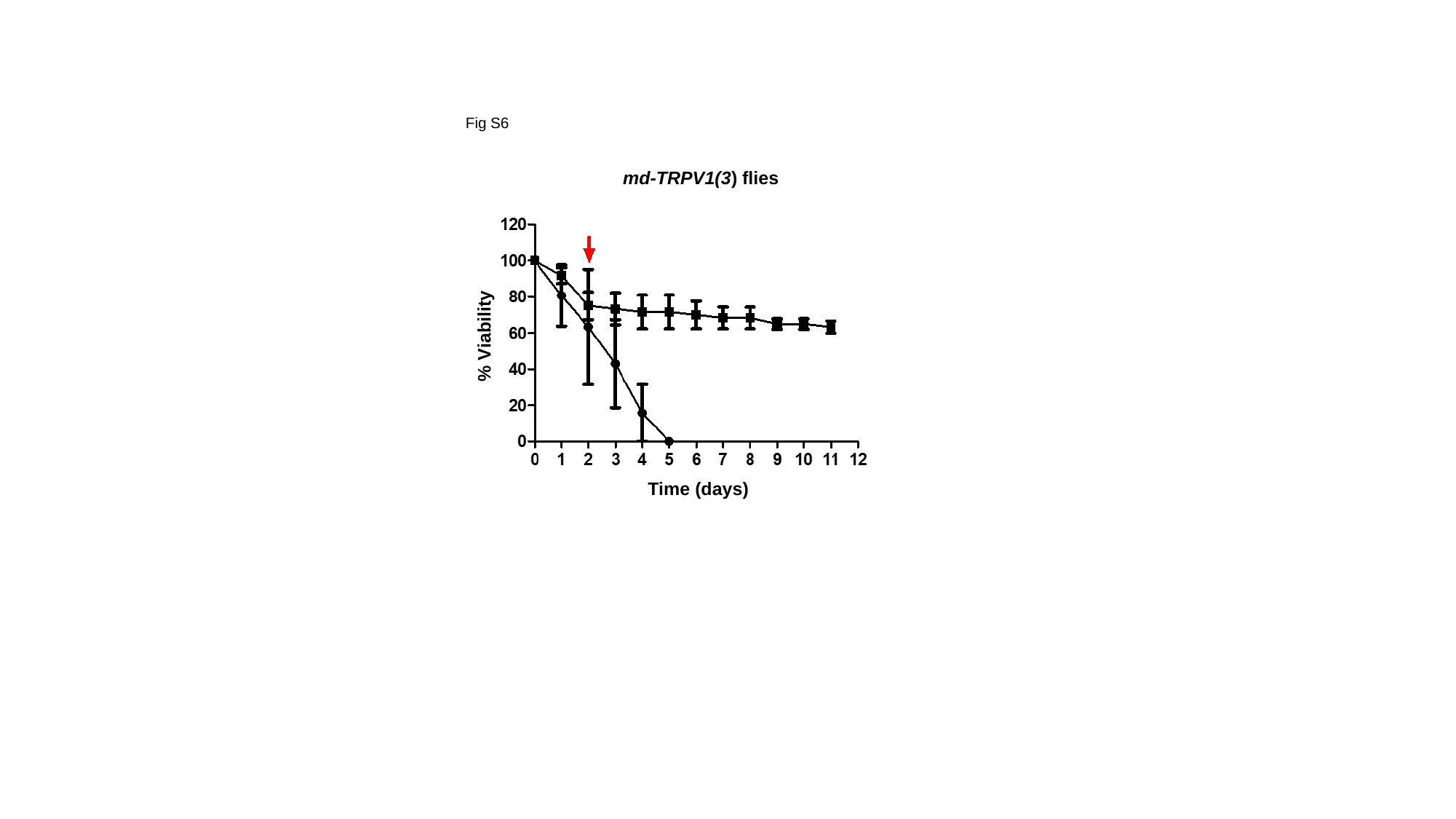

Fig S6
 md-TRPV1(3) flies
% Viability
Time (days)

Supplement: S6 Fig — The viability of md-TRPV1(3) flies reared on capsaicin-containing food (circles) vs that of md-TRPV1(3) flies reared for two days on capsaicin-containing food and then transferred to normal food lacking capsaicin (squares). The day of transfer is indicated by a red arrow. Dots and vertical lines denote means and standard deviations, respectively. n = 60 (20 flies per vial) for each curve. Five-day-old males were used. md-TRPV1(3) denotes one copy of md-Gal4 and three copies of UAS-TRPV1. (PPTX) [file pone.0281874.s008.pptx]
