# Supplementary material for: Drosophila pain sensitization and modulation unveiled by a novel pain model and analgesic drugs
Source: PLoS One. 2023 Feb 16;18(2):e0281874. doi: 10.1371/journal.pone.0281874 (PMC9934396; doi:10.1371/journal.pone.0281874)
Supplement: S3 Fig — Hungry (18 hours starved on water-soaked filters) md-TRPV1(3) flies were offered capsaicin (5 mM)-containing food, on which they exhibited repeated brief sipping (~ one second) and longer recess intervals. The sipping behaviors of three flies over 10 min are shown. md-TRPV1(3) denotes one copy of md-Gal4 and three copies of UAS-TRPV1. Five-day-old males were used. (PPTX) [file pone.0281874.s005.pptx]

## Slide 1
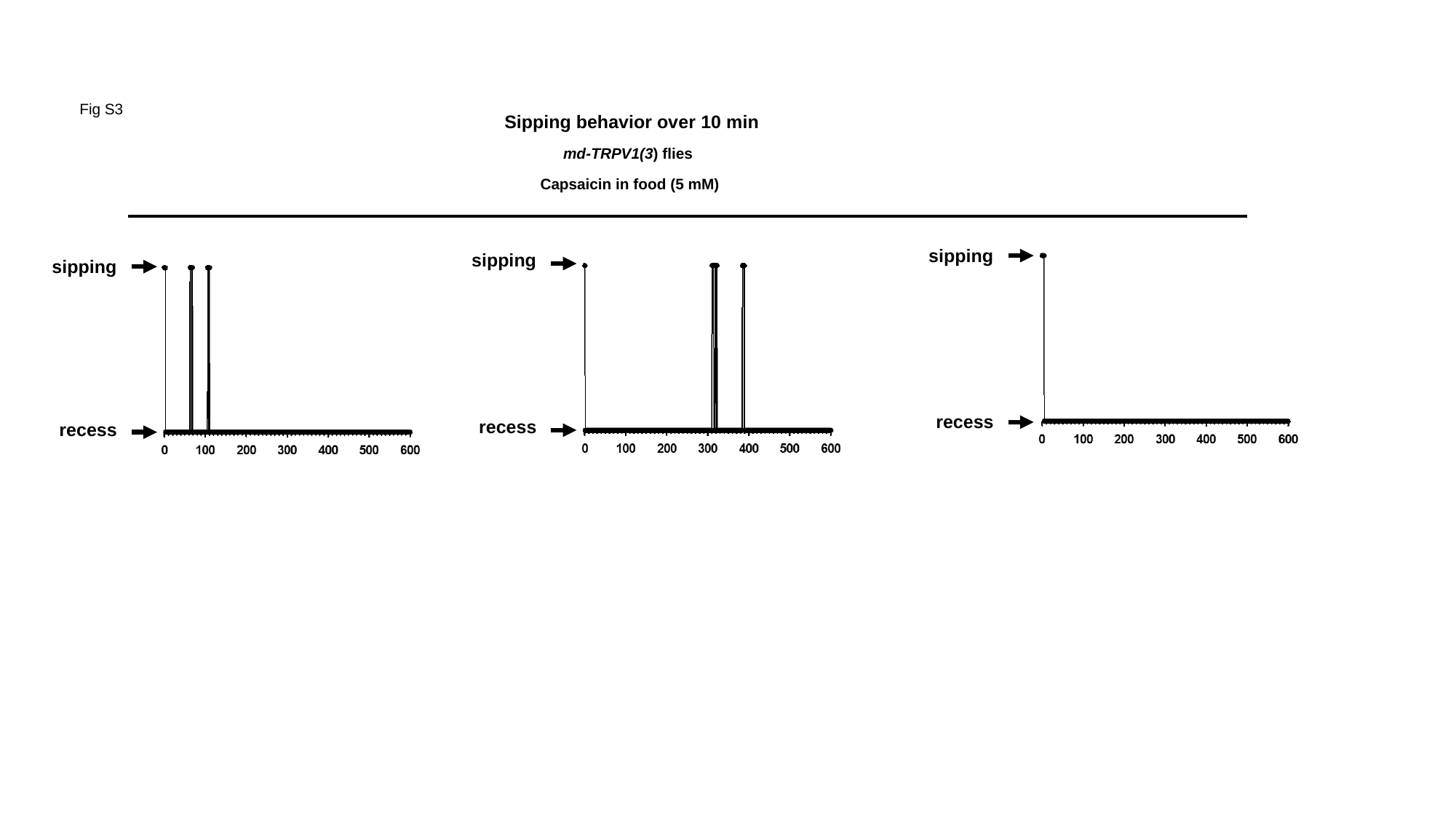

Fig S3
Sipping behavior over 10 min
 md-TRPV1(3) flies
Capsaicin in food (5 mM)
sipping
sipping
sipping
recess
recess
recess
