# Supplementary material for: Drosophila pain sensitization and modulation unveiled by a novel pain model and analgesic drugs
Source: PLoS One. 2023 Feb 16;18(2):e0281874. doi: 10.1371/journal.pone.0281874 (PMC9934396; doi:10.1371/journal.pone.0281874)
Supplement: S4 Fig — Hungry (18 hours starved on water-soaked filters) md-TRPV1(3) flies were offered either capsaicin-containing food (5 mM; left vials) or normal food lacking capsaicin (right vials). Flies given capsaicin-containing food were rarely detectable on the food site, while those provided normal food were frequently detectable on the food site. md-TRPV1(3) denotes one copy of md-Gal4 and three copies of UAS-TRPV1. Five-day-old males were used. (PPTX) [file pone.0281874.s006.pptx]

## Slide 1
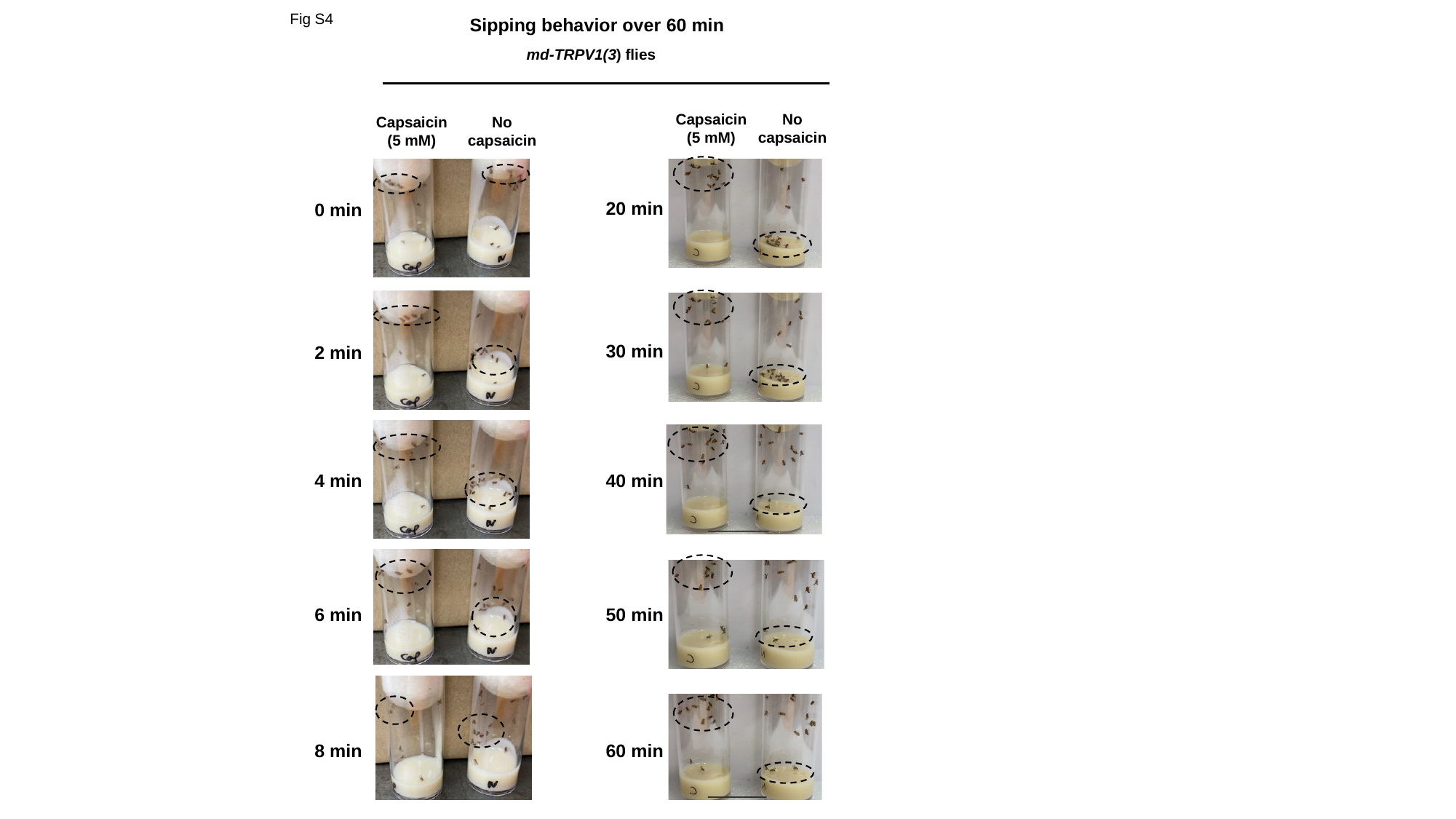

Fig S4
Sipping behavior over 60 min
 md-TRPV1(3) flies
Capsaicin
(5 mM)
No
capsaicin
Capsaicin
(5 mM)
No
capsaicin
20 min
0 min
30 min
2 min
4 min
40 min
6 min
50 min
8 min
60 min
