# Supplementary material for: Drosophila pain sensitization and modulation unveiled by a novel pain model and analgesic drugs
Source: PLoS One. 2023 Feb 16;18(2):e0281874. doi: 10.1371/journal.pone.0281874 (PMC9934396; doi:10.1371/journal.pone.0281874)
Supplement: S5 Fig — (A) Viabilities of md-TRPV1(3) reared on capsaicin-containing food or water-soaked filters at 29°C. md-Gal4 flies were used as control. n = 40 for each experiment. (B) Empty abdomen of a md-TRPV1(3) fly that was provided capsaicin (5 mM) food and full abdomen of one provided normal food for 36 hours at 25°C. n = 10. (C) Weight loss of md-TRPV1(3) flies on capsaicin (5 mM) food. Weight was measured after transfer to normal food or capsaicin (5 mM) food for 21 hours and 28 hours, respectively, at 25°C. n = 40 for each point. Five-day-old males were used. md-TRPV1(3) denotes one copy of md-Gal4 and 3 copies of UAS-TRPV1. (PPTX) [file pone.0281874.s007.pptx]

## Slide 1
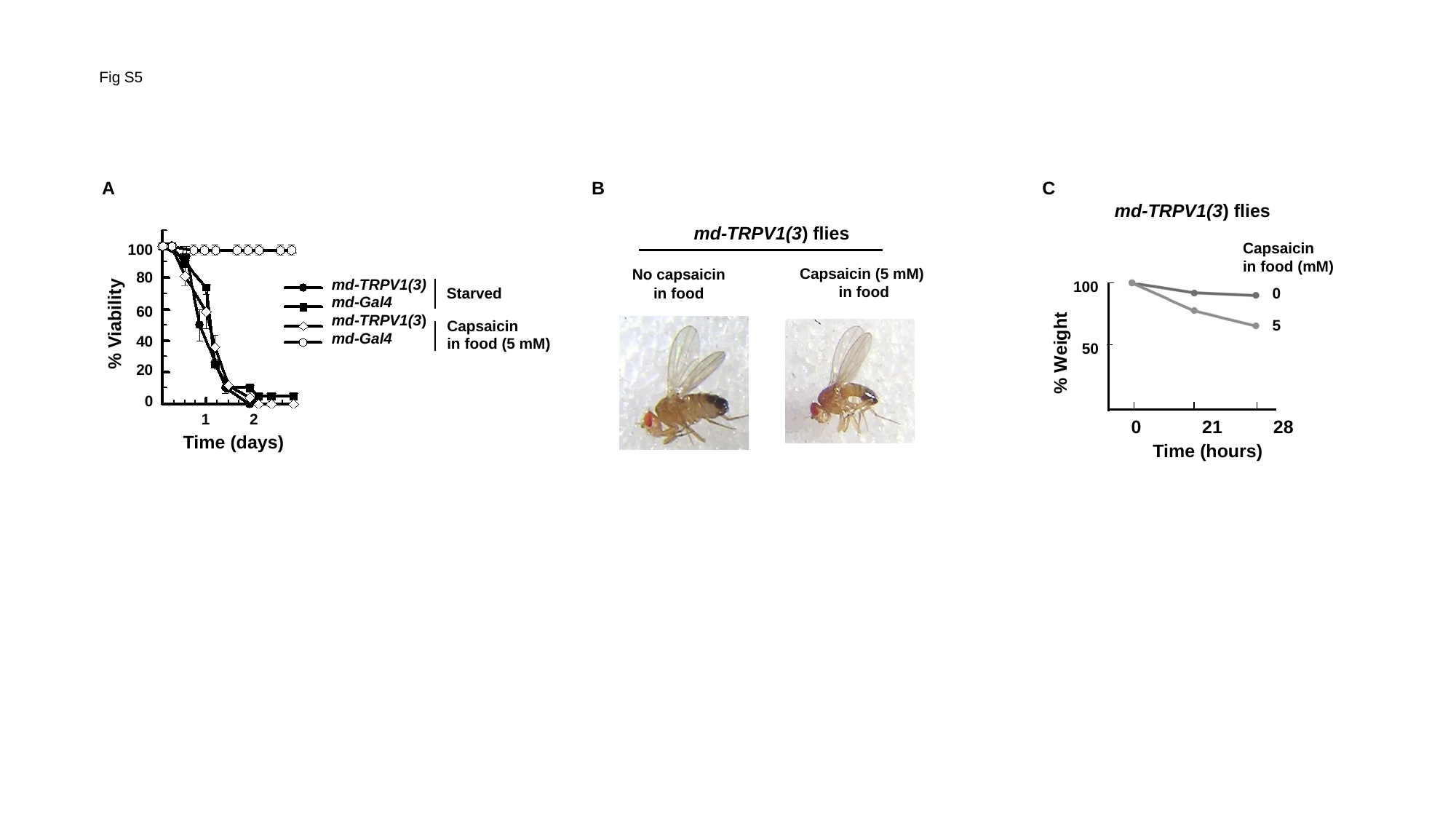

Fig S5
A
B
C
 md-TRPV1(3) flies
 md-TRPV1(3) flies
Capsaicin
in food (mM)
100
Capsaicin (5 mM)
in food
No capsaicin
in food
80
md-TRPV1(3)
md-Gal4
md-TRPV1(3)
md-Gal4
100
Starved
0
60
% Viability
5
Capsaicin
in food (5 mM)
40
50
% Weight
20
0
1
2
0 21 28
Time (days)
Time (hours)
