# Supplementary material for: Drosophila pain sensitization and modulation unveiled by a novel pain model and analgesic drugs
Source: PLoS One. 2023 Feb 16;18(2):e0281874. doi: 10.1371/journal.pone.0281874 (PMC9934396; doi:10.1371/journal.pone.0281874)
Supplement: S7 Fig — The viability of md-TRPV1(3) flies reared on capsaicin (5 mM)-containing food supplemented with varying amounts of drugs marked in the curve is dependent on the concentration of the supplemented drugs. md-TRPV1(3) denotes one copy of md-Gal4 and three copies of UAS-TRPV1. Five-day-old males were used. (PPTX) [file pone.0281874.s009.pptx]

## Slide 1
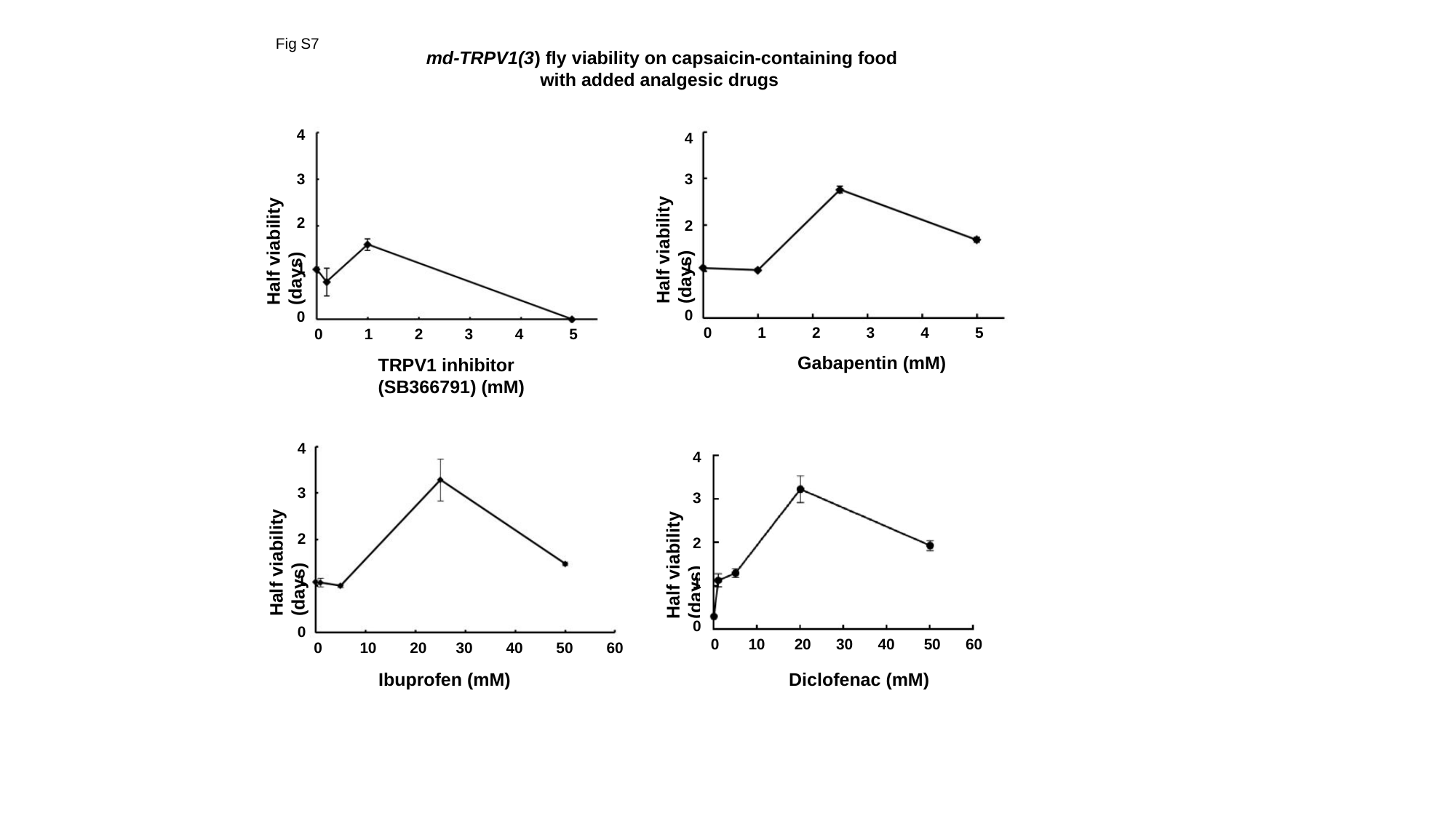

Fig S7
 md-TRPV1(3) fly viability on capsaicin-containing food
with added analgesic drugs
4
4
3
3
Half viability (days)
Half viability (days)
2
2
1
1
0
0
0 1 2 3 4 5
0 1 2 3 4 5
Gabapentin (mM)
TRPV1 inhibitor
(SB366791) (mM)
4
4
3
3
Half viability (days)
Half viability (days)
2
2
1
1
0
0
0 10 20 30 40 50 60
0 10 20 30 40 50 60
Ibuprofen (mM)
Diclofenac (mM)
