# Supplementary material for: Drosophila pain sensitization and modulation unveiled by a novel pain model and analgesic drugs
Source: PLoS One. 2023 Feb 16;18(2):e0281874. doi: 10.1371/journal.pone.0281874 (PMC9934396; doi:10.1371/journal.pone.0281874)
Supplement: S8 Fig — The viability of control (md-Gal4) flies fed normal food supplemented with analgesic drugs (at the concentration most effective in pain reduction) reared at 29°C is shown. Five-day-old males were used. n = 60 (20 flies per vial). The dots and vertical lines denote the means and standard deviations, respectively. (PPTX) [file pone.0281874.s010.pptx]

## Slide 1
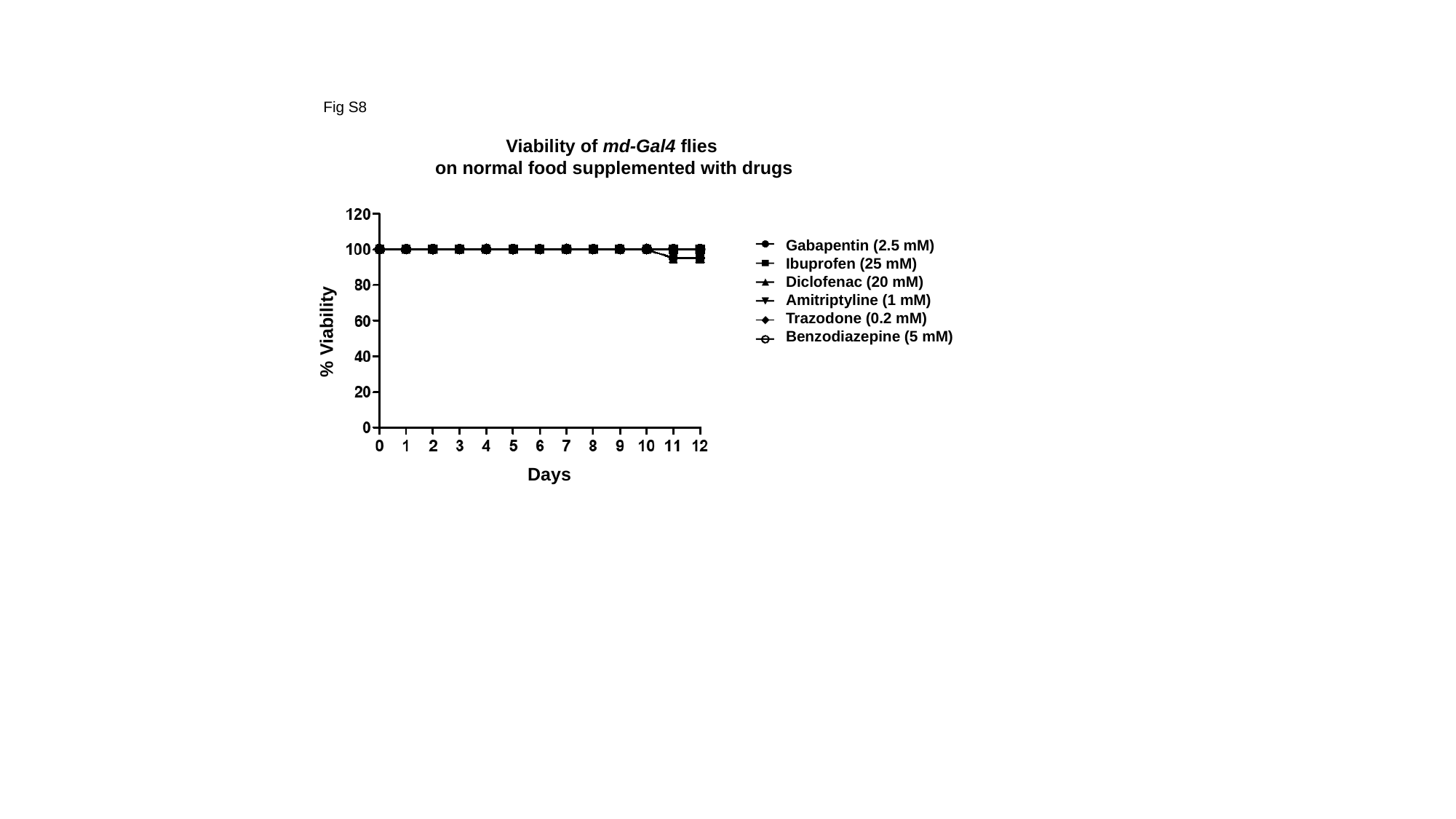

Fig S8
Viability of md-Gal4 flies
 on normal food supplemented with drugs
Gabapentin (2.5 mM)
Ibuprofen (25 mM)
Diclofenac (20 mM)
Amitriptyline (1 mM)
Trazodone (0.2 mM)
Benzodiazepine (5 mM)
% Viability
Days
