# Supplementary material for: Drosophila pain sensitization and modulation unveiled by a novel pain model and analgesic drugs
Source: PLoS One. 2023 Feb 16;18(2):e0281874. doi: 10.1371/journal.pone.0281874 (PMC9934396; doi:10.1371/journal.pone.0281874)
Supplement: S9 Fig — Drugs were added to food at the concentration indicated. Viability of md-TRPV1(3) flies on capsaicin (5 mM) containing food supplemented with non-analgesic drugs at 29°C. Dots and vertical lines denote means and standard deviations, respectively. n = 60 for each curve. Five-day-old males were used. md-TRPV1(3) denotes one copy of md-Gal4 and 3 copies of UAS-TRPV1. (PPTX) [file pone.0281874.s011.pptx]

## Slide 1
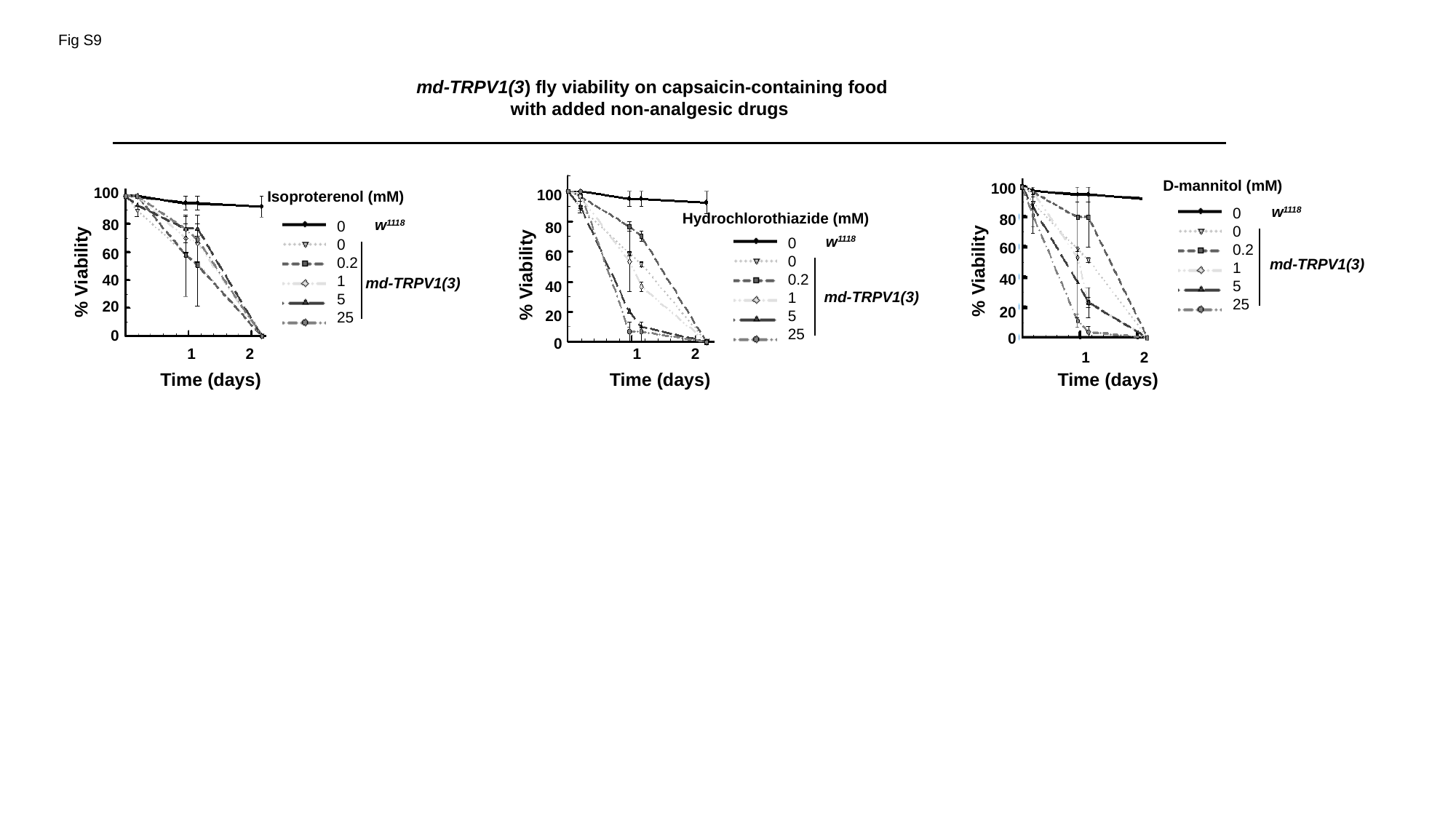

Fig S9
 md-TRPV1(3) fly viability on capsaicin-containing food
with added non-analgesic drugs
D-mannitol (mM)
100
100
100
Isoproterenol (mM)
w1118
0
0
0.2
1
5
25
Hydrochlorothiazide (mM)
80
80
w1118
0
0
0.2
1
5
25
80
w1118
0
0
0.2
1
5
25
60
60
60
md-TRPV1(3)
% Viability
% Viability
 % Viability
40
40
md-TRPV1(3)
40
md-TRPV1(3)
20
20
20
0
0
0
1 2
1 2
1 2
Time (days)
Time (days)
Time (days)
